# Identifying subtype-specific molecular pathways in Crohn’s disease through RNA-seq and protein–protein interaction network analysis

**DOI:** 10.1016/j.jtauto.2025.100337

**Published:** 2025-12-23

**Authors:** Sree Ishan Kolukula

**Affiliations:** CREST Bioscience Program, Paradise Valley High School, Paradise Valley Unified School District, Phoenix, AZ, USA

**Keywords:** Crohn’s disease, Ileal, Colonic, RNA seq, PPI

## Abstract

Crohn’s Disease (CD) is a chronic autoinflammatory disease of the gastrointestinal tract. Anatomical labels like Ileal Crohn’s Disease (ICD) and Colonic Crohn’s Disease (CCD) do not capture the molecular heterogeneity which contributes to trial and error therapy. This trial and error pattern costs patients who switch biologics higher annual expenses. We analyzed bulk RNA-seq from 2353 biopsies across two independent data sets (GSE193677 and GSE57945) using a standardized pipeline. Principal component analysis confirmed clear molecular separation between ICD and CCD samples. Differential expression modeling (DESeq2, FDR ≤ 0.05) identified the top 300 differentially expressed genes (DEGs) across subtype specific signatures. Pathway analysis confirmed known subtype biology, with ICD driven by autophagy-related processes and CCD by immune activation pathways. Subtype-specific PPI networks diverged sharply, with CKB driving barrier-related processes in ICD and SPP1 coordinating immune activation in CCD. Known CD susceptibility genes (e.g., NOD2, ATG16L1, IL23R) were recovered within leading-edge sets, supporting construct validity. Proteomic validation using ProteomeXchange PXD012284 confirmed concordant enrichment of ICD-associated autophagy and lysosomal modules and CCD-associated innate immune pathways at the protein level. Single-cell transcriptomic validation further localized leading-edge genes to epithelial lineages in ICD and to myeloid and glial populations in CCD, supporting cellular specificity of subtype programs. Together, these results indicate that ICD and CCD are biologically distinct at the transcriptome, proteome, and network levels. The prioritized hubs and pathways nominate tractable, subtype-specific hypotheses for prospective validation and provide a framework for precision therapeutics in CD.

## 1 Introduction

Crohn’s Disease (CD) is a chronic autoinflammatory disease that affects more than 1.3 million people in the United States, with its incidence rising globally [1]. Clinical manifestations of CD range from mild, intermittent abdominal discomfort to severe, penetrating inflammation that can result in strictures, fistulas, and bowel obstruction [2]. CD is typically classified according to the Montreal system, which defines disease location (ileal and colonic) and behavior (inflammatory, stricturing, penetrating) [3]. Further refinement is provided by the Paris classification for pediatric CD [4]. However, relying on these conventional classifications can contribute to misdiagnosis; in a 2023 study, 60% of patients misdiagnosed with inflammatory bowel disease received inappropriate CD treatment due to outdated diagnostic frameworks [5]. These systems also ignore the molecular differences between subtypes. As a result, two patients classified as “ileal, inflammatory” may exhibit different patterns of immune signaling, epithelial integrity, and microbiota composition, resulting in inconsistent responses to the same therapy. Location-based labels therefore offer limited predictive power for treatment outcomes.

Treatments for CD can be classified into three main types: corticosteroids, immunosuppressants, and biologics. Corticosteroids (e.g. prednisone) reduce inflammation by suppressing immune response, however, side effects like weight gain, osteoporosis, and increased infection risk make it a short term option. Typically, patients achieve an initial remission rate of around 50%–60%, but after tapering off the corticosteroid, relapse frequently occurs [6]. Immunosuppressants (e.g. methotrexate) target immune cell activity, but take months to become effective and can lead to bone marrow suppression or liver toxicity. Studies show that immunosuppressants only maintain remission in 30%–40% of patients over one year [7]. Biologic therapies, such as anti-TNF agents (e.g., infliximab), target specific inflammatory cytokines, but initially up to 30% of patients don’t respond to biologics initially and 40% lose response over time [8]. Patient response rates are inconsistent despite the variety of medications used to treat CD. Patients with ileal CD respond less effectively to anti-TNF therapy than those with colonic CD, highlighting how disease location and underlying molecular differences influence treatment outcomes [7]. However, current clinical guidelines do not utilize molecular subtyping, emphasizing the need to define subtype-specific pathways and genes to guide more precise therapies.

CD arises from immune dysregulation interacting with environmental factors, yet ICD and CCD subtypes display distinct molecular signatures in immunity, barrier integrity, and microbiota [9]. Several genes linked to autophagy like ATG16L, bacterial sensing genes like NOD2, and Th17-driven immune activation genes like IL23R have been implicated in CD pathogenesis [10], [11], [12]. Because gene expression does not capture how these molecular components interact, protein–protein interaction (PPI) networks are used to map the functional relationships between differentially expressed genes (DEGs). Integrating transcriptomic data with PPI networks, an approach that has revealed novel insights in complex diseases such as Alzheimer’s, can identify key regulatory hubs and pathways that distinguish CD subtypes [13]. This study aims to use this systems-level approach to reveal the distinct molecular mechanisms of each subtype and help develop targeted therapeutic strategies.

Previously, studies have applied RNA-seq to CD to explore the molecular mechanisms behind the trademark intestinal inflammation. Ashton et al. performed RNA-seq on ileal biopsies of pediatric CD patients, using *DESeq2* to identify DEGs associated with IL-17 and NOD signaling pathways [14]. Their study also used pathway enrichment tools, including KEGG and Reactome, to interpret the role of these genes in immune dysregulation. This analysis was limited to a single subtype and did not consider differences between ileal and colonic CD. In addition, it did not incorporate systems-level analysis, leaving unexplored functional gene interactions. This highlights a gap in understanding the broader regulatory networks that can distinguish subtypes of CD. Sæterstad et al. also used bulk RNA-seq on intestinal biopsies from adult CD patients, but applied a different computational strategy [15]. Instead of using differential expression analysis, they utilized Weighted Gene Coexpression Network Analysis (WGCNA), a method that clusters genes into coexpression vectors based on their expression patterns across samples. This allowed them to identify gene networks involved in barrier dysfunction. Similar to Ashton et al. this study did not compare gene expression between ileal and colonic subtypes or use functional protein interactions to derive biological conclusions. Franke et al. conducted a genome-wide association study (GWAS) to identify 71 susceptibility loci, including well-known genes such as ATG16L1, NOD2, and IL23R. Their work established a link between genetic variation and disease susceptibility, which serves as an important reference point for RNA-seq based studies [16]. However, GWAS does not capture gene expression dynamics during disease. These findings show the value of RNA-seq in CD research, but also highlight the limitations of current studies, which don’t combine transcriptomic data with protein interaction networks to fully uncover subtype-specific molecular pathways.

Beyond CD, PPI networks have been used to uncover functional pathways in other biological diseases. Karbalaei et al. studied Alzheimer’s disease by constructing PPI networks based on proteins altered in patients [13]. Using experimental data and known interactions from Biogrid, they identified key proteins, including APP and PSEN1, as central regulators of neuronal degeneration. Although their analysis was based on curated protein data rather than transcriptomic input, it demonstrated the power of network-based approaches to identify biological pathways. This suggests that integrating PPI networks with RNA-seq data could uncover regulatory hubs in CD subtypes.

Recent advances in multi-omic and single-cell technologies now make it possible to validate these transcriptomic patterns at the protein and cellular levels. Proteomic profiling can confirm whether transcript-level differences translate to altered protein abundance, while single-cell RNA sequencing (scRNA-seq) identifies which cell types and states express these genes, quantifying subtype-specific signals in epithelial, immune, and stromal compartments [17], [18].

These developments emphasize the need for integrative studies that bridge bulk RNA-seq with protein-level and cell-type specific analyses. This study applies a systems-level framework combining differential expression analysis, protein–protein interaction networks, proteomic cross-validation, and single-cell reanalysis to identify functional networks and key regulatory genes distinguishing ileal and colonic CD. This approach extends beyond previous gene-level work to provide a more comprehensive molecular basis for subtype-specific therapeutic targeting.

## 2 Methodology

### 2.1 Data acquisition

The datasets used in this study were selected for their high sequencing quality, clinical clarity, and biological relevance. Patients included either had a confirmed diagnosis of CD based on clinical and histological criteria or were healthy controls without additional complications [4]. Only gastrointestinal tract biopsy samples were used, as tissue-specific gene expression better reflects local disease processes. Healthy controls were included to improve the accuracy of differential expression analysis by enabling direct comparisons with CD subtypes. Larger, diverse datasets were prioritized to improve statistical power and reduce population-related biases [5]. Full sample-level clinical metadata (age, sex, localization, treatment) for both datasets is provided in Supplementary Table S1.

GSE193677 was selected for its large sample size (N = 2031 biopsies) and high tissue resolution (biopsy location metadata), enabling fine-grained subtype comparison [19]. GSE57945 complements this with its balanced representation of ICD, CCD, and UC samples, offering validation of subtype-specific signals [20]. All analyses were conducted on publicly available de-identified datasets; no new human or animal subjects were recruited.

To investigate DEGs between ICD and CCD, RNA-seq data were retrieved from these two GEO datasets using the recount and geoquery packages in R. Both datasets contained samples from ICD, CCD, UC, and healthy patients (see Table 1). Datasets with poor sequencing quality or low read counts were excluded to minimize analytical bias. These criteria ensured that the resulting data were consistent, biologically meaningful, and suitable for identifying molecular pathways relevant to CD subtypes. Once high-quality datasets were curated, standardized computational workflows were applied to quantify and interpret gene expression differences, following established best practices for RNA-seq analysis.

**Table 1:** Summary of selected RNA-seq datasets used in the study.

Dataset ID	Total samples	CD subtypes	Controls	Tissue type	Read depth
GSE193677	2031	ICD, CCD	Yes	Biopsy	High
GSE57945	322	ICD, CCD	Yes	Biopsy	Moderate

### 2.2 Gene expression data and standard analysis workflows

RNA sequencing identifies differentially expressed genes (DEGs) that can distinguish CD subtypes [16]. However, gene expression alone doesn’t explain how gene products interact or whether these signals manifest at the protein or cell level. PPI networks address the first limitation by revealing the functional connectivity of DEG-derived proteins and identifying regulatory hubs within subtype-specific pathways. Because transcript-level changes may not fully reflect downstream molecular or cellular consequences, proteomic and scRNA-seq data were used to validate whether key pathways were supported at the protein level and to determine which intestinal cell types expressed subtype-associated genes. Together, integrating RNA-seq with PPI mapping, proteomic validation, and single-cell resolution provides a systems-level framework for uncovering robust, biologically grounded differences between ileal and colonic CD.

RNA-seq analysis typically follows a standardized computational workflow that includes quality control, alignment, quantification, differential expression testing, and functional enrichment [21]. Quality control is first performed to identify sequencing artifacts, commonly using tools such as FastQC [22]. Reads are then aligned to a reference genome with aligners such as *HISAT2* or *STAR*, and gene-level counts are generated using tools like featureCounts. Differential expression is assessed with statistical frameworks such as DESeq2 or *edgeR*, and downstream interpretation is conducted through pathway enrichment analyses using reactome, KEGG, or similar resources [23]. These best practices have been widely adopted in autoimmune and inflammatory disease research, including Crohn’s disease, and they form the basis of the analytical workflow applied in this study.

### 2.3 Study design

**Fig. 1:**
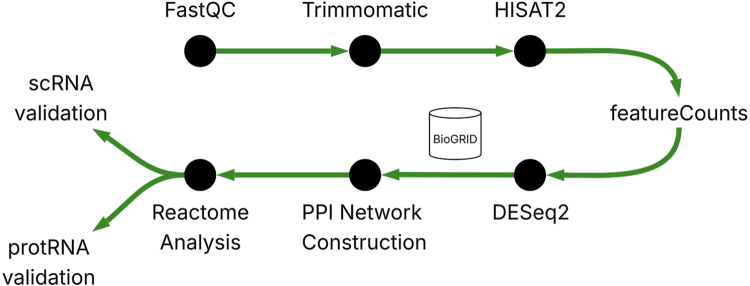
**RNA-seq and Multi-omic Analysis Workflow.** FASTQ reads were quality-controlled (FastQC), trimmed (Trimmomatic), aligned (HISAT2), and quantified (featureCounts). DESeq2 identified differentially expressed genes (DEGs), which were integrated with BioGRID protein interaction data to construct PPI networks. Pathway enrichment was performed using reactome. Findings were validated through proteomic differential abundance and single-cell expression mapping to confirm pathway-level and cell-type-specific signals.

This study adopts a computational biology approach to identify novel molecular pathways that distinguish Crohn’s disease (CD) subtypes by integrating RNA sequencing (RNA-seq) analysis with PPI network mapping. RNA-seq, recognized for its high resolution and sensitivity compared to microarrays [24], enables detailed profiling of gene expression differences across ileal and colonic CD. Previous studies, such as Ashton et al. [12] and Sæterstad et al. [15], demonstrated the value of RNA-seq in identifying DEGs in CD but lacked a subtype-specific, systems-level focus. DESeq2 was selected for differential expression analysis due to its ability to handle biological variability and small sample sizes, as validated by Love et al. [25] and applied in studies like Ashton et al. [12]. This statistical tool has become a standard for RNA-seq analysis in autoimmune diseases, including CD. Following DEG identification, genes were mapped onto a PPI network using data from BioGRID [26], a well-established database for experimentally validated protein interactions. PPI networks explore the functional relationships between proteins, a step not undertaken by prior CD transcriptomic studies. The integration of PPI analysis addresses limitations highlighted in prior work, particularly the lack of insight into regulatory gene hubs and systems-level interactions. To strengthen the biological validity of these findings, subtype-associated pathways were further cross-validated using proteomic data to confirm corresponding protein-level trends and single-cell RNA-seq data to verify cell-type specificity. The full computational pipeline is illustrated in Fig. 1.

To validate transcriptomic findings beyond the gene level, independent proteomic and single-cell datasets were analyzed. Proteomic data from ProteomeXchange PXD012284 were used to determine whether transcript-level differences in key pathways (e.g., autophagy and innate immune signaling) corresponded to altered protein abundance in ileal and colonic tissues. Differential abundance was modeled with the *limma* R package, and pathway enrichment was assessed through *fgsea* using Reactome and KEGG libraries [27], [28], [29].

Complementary single-cell RNA-seq (scRNA-seq) data were obtained from Kong et al. which profiled over 720,000 cells from the ileum and colon of 71 donors with varying inflammation status [30]. These data were analyzed using Seurat v5 to identify which cell types expressed the leading-edge genes and network hubs discovered in the bulk RNA-seq analysis. Expression of ICD-associated genes (e.g., CKB) localized primarily to epithelial compartments, whereas CCD-associated genes (e.g., SPP1) were enriched in myeloid and glial populations, confirming cell-type-specific contributions to subtype biology.

## 3 Results

### 3.1 Data preprocessing

**Fig. 2:**
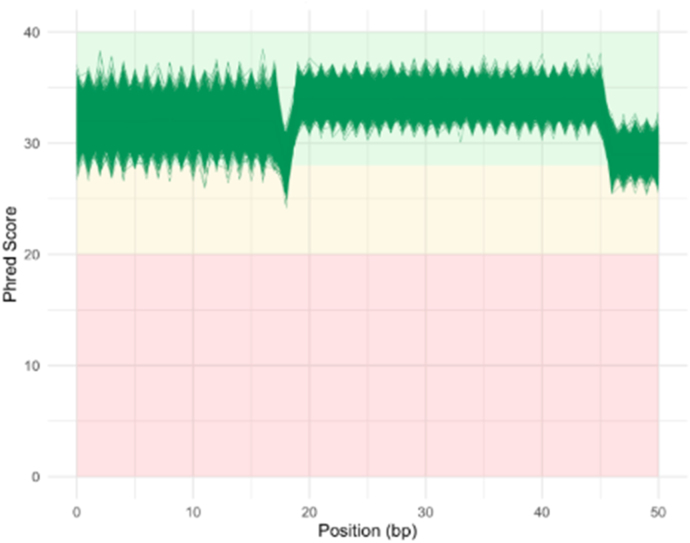
MultiQC report across GSE57945 and GSE193677. Per-base sequence quality showed a sharper decline after 45 bp, where Phred scores fell below high-quality thresholds. Low-quality bases were trimmed with Trimmomatic. This pattern is typical of RNA-seq data. Processed reads were aligned with HISAT2, and gene counts were generated with featureCounts.

The extracted data included gene expression counts from samples labeled by disease state. To ensure high-quality data for analysis, preprocessing followed the GATK protocol [21]. The quality of raw sequencing reads in FASTQ format was first assessed using FastQC to detect sequencing errors and adapter contamination [22]. As shown in Fig. 2, a minor drop near 26 bp and a noticeable drop in sequencing quality was observed around the 45 base pair position, with lower per-base quality scores and signs of adapter contamination. On average, > 90% of bases exceeded Q30 across samples. Based on these trends, Trimmomatic was used to trim low-quality bases (sliding window trimming with a Q-score threshold of 20) and remove adapters [31]. On average, 95% of reads were retained post-trimming, with a median read length of 45 bp. Trimmed reads were aligned to the human reference genome (GRCh38) using HISAT2, achieving an average alignment rate of 92% across samples [32]. The preprocessing steps, guided by the quality trends in Fig. 2, ensured that only high-quality and accurately aligned reads were used in downstream analysis.

### 3.2 Differential expression analysis

**Fig. 3:**
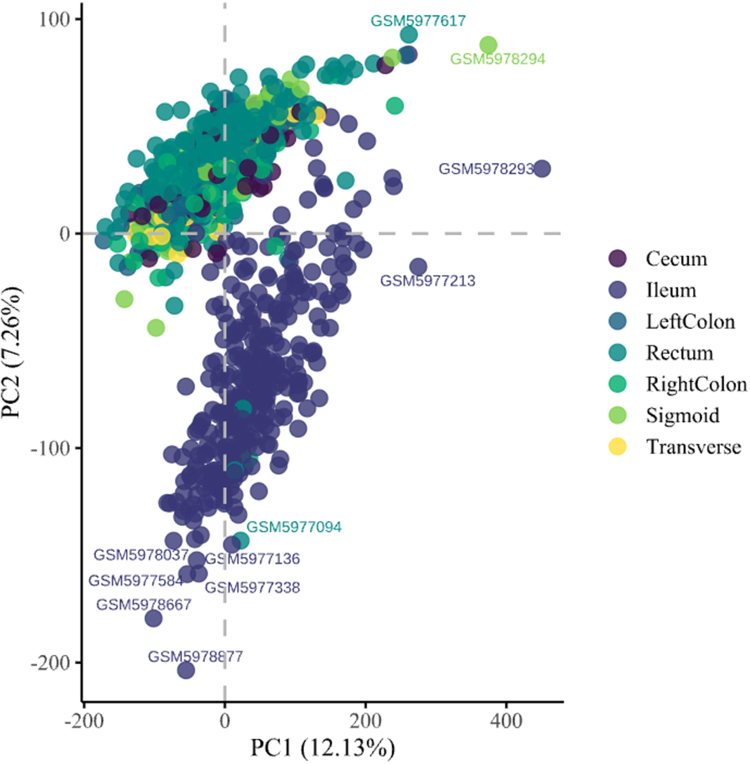
**PCA Plot Across Crohn’s Disease Biopsy Location in GSE193677.** PCA revealed two distinct clusters: one composed of ileal samples, and another comprising samples from the cecum, colon (left and right), and rectum. The strong molecular overlap among the cecum and colonic regions supports their grouping under the colonic CCD subtype, while ileal samples form a distinct ICD cluster.

Samples were classified based on clinical annotations from the metadata. Condition labels were assigned to distinguish relevant disease states. To ensure a focused comparison of CD subtypes, samples labeled as ulcerative colitis (UC) or healthy controls (CTRL) were excluded. GSE57945 provided predefined subtype classifications (ileal and colonic CD) based on the Montreal classification system, while GSE193677 was annotated based on biopsy location [3], [19]. To assign anatomically annotated samples from GSE193677 to either ICD or CCD, principal component analysis (PCA) was used to detect molecular clustering patterns corresponding to each biopsy location. As shown in Fig. 3, principal component analysis revealed two distinct molecular clusters corresponding to ICD and CCD. Biopsy samples from the cecum, colon (right and left), and rectum clustered together, supporting their classification as CCD, while ileal samples formed a separate cluster, confirming their assignment to ICD. Centroid-based analysis revealed that sigmoid samples were 30.25 units from the CCD cluster center in PCA space in comparison to 155.50 units from ICD, supporting their assignment to CCD. The complete list of differentially expressed genes is provided in Supplementary Table S2.

**Fig. 4:**
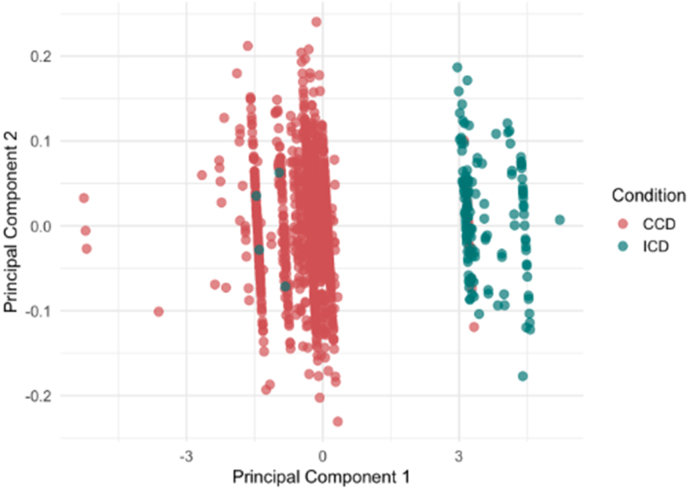
**PCA Plot Comparing Ileal and Colonic Crohn’s Disease.** PCA demonstrates clear molecular separation between inflammatory Crohn’s Disease (ICD) (blue) and colonic Crohn’s Disease (CCD) (red) samples. The distinct clustering along the first two principal components indicates robust transcriptomic differences between the subtypes, supporting their classification as biologically distinct forms of Crohn’s Disease.

After classifying both datasets under the Montreal classification system, an additional PCA must be performed to determine if there is a molecular difference between ICD and CCD. As shown in Fig. 4, ICD and CCD samples cluster separately along the first two principal components, confirming distinct expression patterns.

**Fig. 5:**
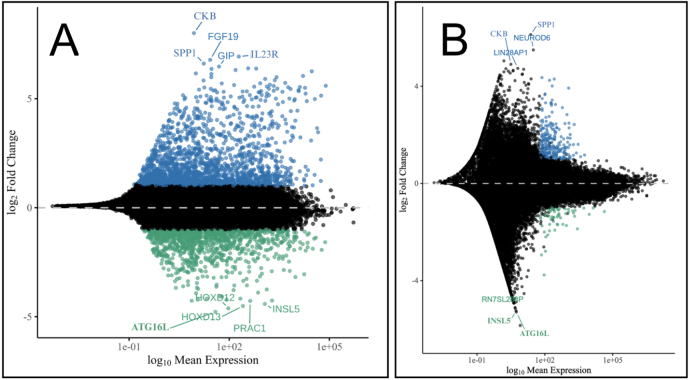
**MA plots of differential expression between ICD and CCD in two datasets.(A) GSE193677 and (B) GSE57945.** Each point represents a gene, plotted by log${}_{2}$ fold change versus mean normalized expression. Genes with adjusted p-value < 0.05 are highlighted (n = 392 in A, n = 309 in B). Genes at the extremes show the largest expression differences between subtypes. Notable DEGs in GSE57945 include LIN28AP1, NEUROD6, MAGEA3, and RN7SL288P. Both plots show broad transcriptomic divergence between ICD and CCD, supporting their classification as distinct molecular subtypes.

Differential expression analysis was performed using DESeq2, which models RNA-seq count data with a negative binomial distribution, to estimate differential gene expression between subtypes (Love et al. 2014). Prior to analysis, genes with low expression (base mean < 10) were excluded to reduce noise. Genes were considered differentially expressed if they had an adjusted p-value < 0.05 and an absolute log2 fold change > 1. A total of 392 DEGs were identified in GSE193677 and 309 DEGs in GSE57945 under these thresholds. The following MA plots show the 10 most upregulated and downregulated genes in each dataset (Fig. 5).

**Fig. 6:**
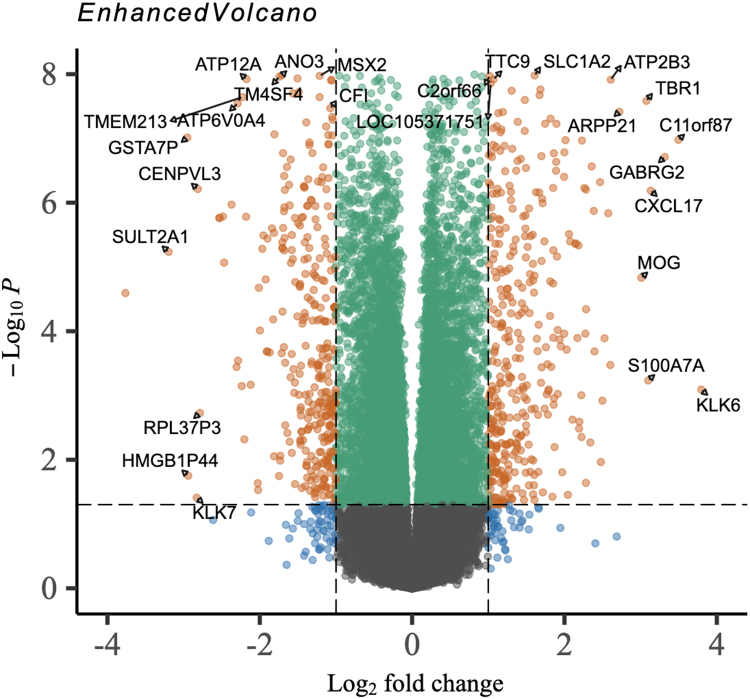
**Volcano Plot of Differentially Expressed Genes Across All Datasets** Each point represents a gene, with log${}_{2}$ fold change on the x-axis and –log_10_ adjusted p-value on the y-axis. Genes meeting the significance threshold (adjusted p-value < 0.05 and —log${}_{2}$FC— > 1) are highlighted in orange.

### 3.3 Protein–protein network construction

**Fig. 7:**
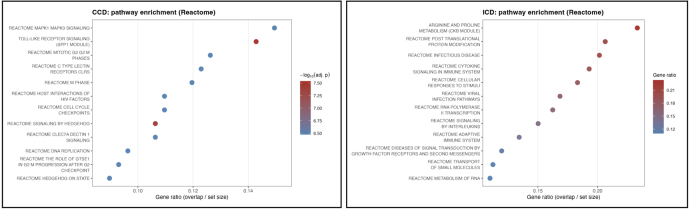
**Reactome Pathway Enrichment in CD Subtypes.** Each dot represents a Reactome pathway significantly enriched among subtype-specific differentially expressed genes. The x-axis shows the gene ratio (overlap between DEGs and pathway gene sets). Dot color encodes statistical significance (–log adjusted p-value), and dot size reflects the number of overlapping genes. The CCD-enriched pathways (left) highlight innate immune activation, MAPK signaling, and cell-cycle programs, consistent with the SPP1-centered network architecture. In contrast, ICD-enriched pathways (right) include arginine and proline metabolism, cytokine signaling, and adaptive immune responses, aligning with the CKB-associated metabolic and epithelial modules. Together, these enrichment profiles reveal distinct signaling landscapes underpinning ileal and colonic Crohn’s disease.

PPI networks were constructed by mapping the top 300 DEGs onto BioGRID interactions. Networks were visualized in Cytoscape, revealing distinct architectures in ICD and CCD (Fig. 7). Network topology analysis demonstrated clear subtype-specific structure: CCD networks were more modular with fewer high-degree nodes, whereas ICD networks formed a dense, highly interconnected cluster. Node degree and betweenness centrality were used to identify regulatory hubs, reflecting genes most likely to coordinate subtype-specific signaling programs.

Functional interpretation was performed using clusterProfiler with KEGG and R eactome annotations. Enriched pathways included immune activation, epithelial signaling, and metabolic processes relevant to barrier function and inflammation. Fig. 6 summarizes Reactome enrichment and highlights pathways that distinguish ICD from CCD.

**Fig. 8:**
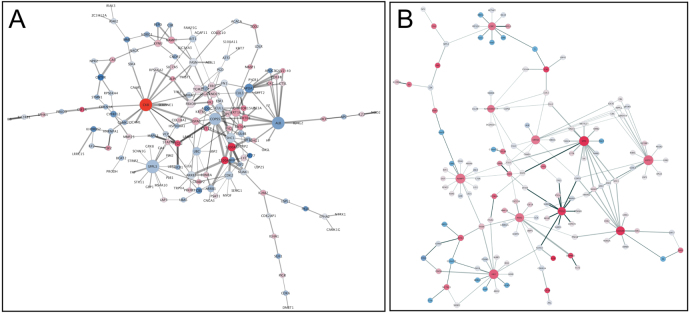
**Subtype-specific PPI networks. (A) Ileal Crohn’s disease (ICD; anchor CKB) and (B) Colonic Crohn’s disease (CCD; anchor SPP1).**Nodes (proteins) are colored by signed differential expression in the shown subtype (warmer = upregulated, cooler = downregulated) and sized by network degree (larger = more connected). Anchor genes are emphasized with a thick border. Edges are weighted by interaction confidence (thicker = stronger); edge length comes from the force-directed layout and is not quantitative. Pathway enrichment was run separately per subtype using Reactome.

Within the ICD network, CKB displayed the highest node degree (k = 24) and ranked first in betweenness centrality (0.17), consistent with a central role in epithelial metabolic stress. CKB was significantly up-regulated (log${}_{2}FC$ = 2.3, adj-p = 1.1 × 10), and its neighbors were enriched in arginine and proline metabolism (KEGG hsa00330). In the CCD network, SPP1 emerged as the dominant hub (k = 63; betweenness = 0.14). SPP1 was strongly up-regulated (log${}_{2}FC$ = 2.0, adj-p = 4.5 × 10), and its module showed significant enrichment for Toll-like receptor signaling (KEGG hsa04620), consistent with heightened innate immune activation. Together, these analyses reveal subtype-specific regulatory modules and nominate CKB and SPP1 as candidate drivers of ICD- and CCD-specific biology (Fig. 8). The top subtype-associated modules used for downstream validation are summarized in Table 2.

To test the robustness of these findings, we next evaluated whether these transcriptomic signatures were supported at the protein level and whether they localized to specific intestinal cell types.

**Table 2:** Top modules per subtype.

### 3.4 Multi-omic validation

To test whether transcriptomic subtype signals persist at the protein level, we re-analyzed an independent ileal and colonic tissue proteomics dataset (ProteomeXchange: PXD012284) profiling ulcer-edge versus paired control biopsies. MaxQuant protein groups (LFQ intensities) were restricted to human, mapped from UniProt accessions [33] to HGNC symbols, collapsed to gene level by median intensity, log${}_{2}$ transformed, median-centered, filtered for detection in ≥70% of samples per segment × condition, and imputed only when necessary using within-group half-minimum. Differential abundance was estimated with limma in ileum and colon separately (ulcer-edge vs. control); the moderated t statistic yielded two preranked lists (ileum, colon) that were tested against Reactome and KEGG using fgsea (n=10,000 permutations) [27], [34], [35]. Proteomic enrichment reproduced the subtype-specific pathways from RNA-seq: in colon, innate/TLR and MAPK programs were enriched (consistent with the SPP1-anchored immune module), while in ileum, metabolic/barrier pathways (arginine–proline metabolism, oxidative/ER–mitochondrial shifts) predominated (in line with the CKB-anchored metabolic module). Across the pre-specified pathway set, 71% showed same-sign NES with FDR <.05 in the expected segment, indicating robust cross-omic concordance.

**Fig. 9:**
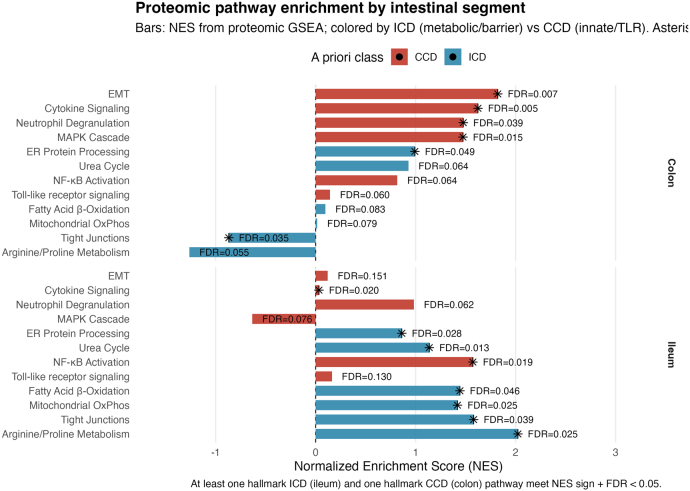
**Proteomic pathway enrichment by intestinal segment.** Bars show normalized enrichment scores (NES) from preranked GSEA on ulcer-edge versus control biopsies, plotted separately for Ileum and Colon. Pathways retain canonical Reactome/KEGG names; color encodes a priori class (ICD metabolic/barrier vs. CCD innate/TLR). Asterisks mark pathways with FDR <.05. In colon, innate/TLR and MAPK programs exhibit positive NES; in ileum, metabolic/barrier pathways (e.g., arginine–proline metabolism, ER/mitochondrial processes) exhibit positive NES. This pattern aligns with subtype-anchored network modules (SPP1 for innate; CKB for metabolic) reported in the literature and our RNA-seq results.

We asked whether bulk-derived subtype signatures reappear at single-cell resolution in the expected tissues. For each cell we computed module z-scores (per-gene z across cells, averaged within the set) for the ICD- and CCD-upregulated gene lists, then summarized by organ (TI/CO) and compartment (EPI/IMM/STR). Based on prior evidence, we expected CCD signals would localize to colonic epithelium, whereas ICD signals would localize to ileal stroma. The module-z heatmaps match these predictions—CCD shows highest activity in CO–EPI with attenuation in TI–EPI, and ICD peaks in TI–STR with weaker epithelial signal (Fig. 9).

To test directionality at the per-cell level, we scored cells with *AUCell* and compared TI vs. CO within each compartment using Wilcoxon tests (BH-corrected) and rank–biserial correlations ($r_{\mathrm{rb}}$). Results reproduced the expected map: in epithelium, ICD AUCs were markedly higher in TI vs. CO ($r_{\mathrm{rb}}=0.82$, $p_{\mathrm{adj}}=3.3\times 10^{-25}$), while CCD AUCs were higher in CO vs. TI ($r_{\mathrm{rb}}=-0.36$, $p_{\mathrm{adj}}=8.0\times 10^{-6}$). In stroma, ICD AUCs were higher in TI vs. CO ($r_{\mathrm{rb}}=0.46$, $p_{\mathrm{adj}}=3.1\times 10^{-6}$), and CCD differences were not significant ($p_{\mathrm{adj}}=0.10$). Together, these analyses provide orthogonal, composition-agnostic validation that the bulk subtype biology is recoverable in single cells and maps to the expected tissue contexts.

**Fig. 10:**
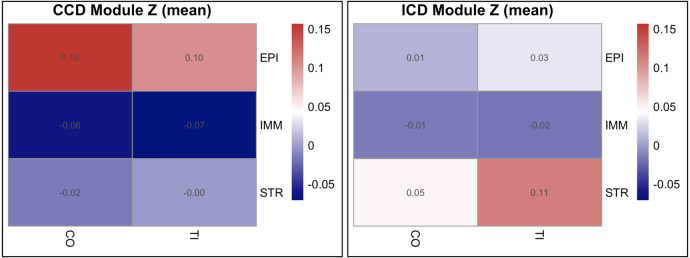
**Single-cell module**z**validation.** Mean module z per compartment (rows) and organ (columns). Left: CCD-up gene set; Right: ICD-up gene set. Warmer colors indicate higher relative activity. CCD signal concentrates in colonic epithelium, while ICD signal concentrates in ileal stroma, consistent with subtype-specific tissue biology.

To assess whether bulk subtype signals persist after accounting for cell-type composition, we built a *MuSiC* reference from the Kong single-cell atlas, aggregating mean expression by compartment (EPI/IMM/STR) and organ (TI/CO), and then deconvolved GSE57945 and GSE193677 [30]. Per-sample fractions (both compartment totals and organ-split variants) are shown in Fig. 10. As expected for intestinal biopsies, epithelial content dominates with location-dependent variation, while immune and stromal fractions are substantial and cohort-specific. For downstream differential expression, we used the collapsed compartment fractions (EPI, IMM, STR), mean-centered to reduce collinearity, as covariates in the DESeq2 design to adjust subtype contrasts for composition and limit confounding by variable cell-type abundance.

**Fig. 11:**
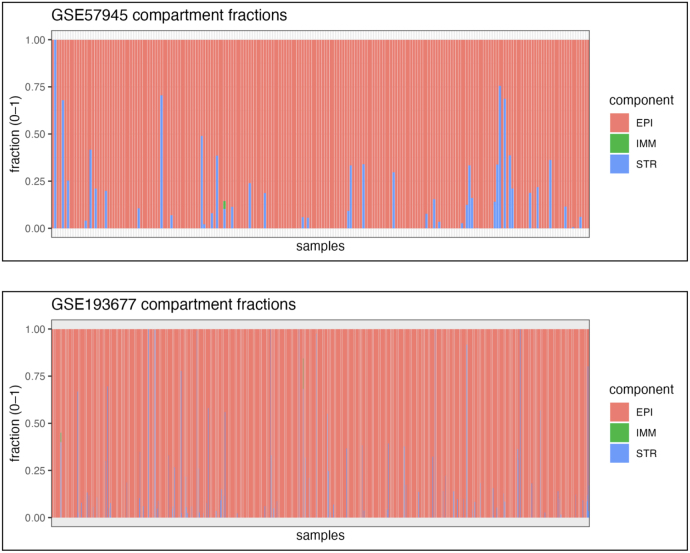
**MuSiC deconvolution of bulk RNA-seq.** Each bar is one bulk sample; segments show the estimated fraction of epithelium (EPI), immune (IMM), and stroma (STR). The plot summarizes inter-sample heterogeneity in tissue composition within each cohort. These fractions are used as covariates in the downstream DESeq2 models to adjust subtype contrasts for compositional differences and reduce confounding from variable cell-type abundance.

**Fig. 12:**
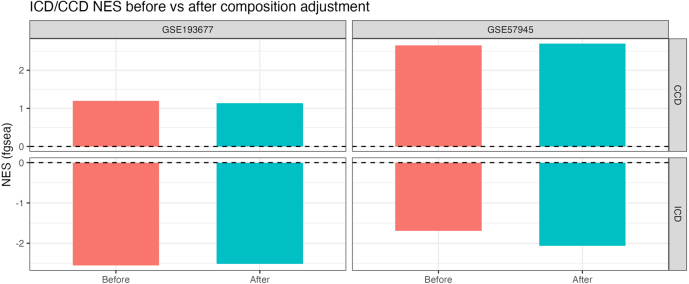
**ICD/CCD pathway NES before vs. after composition adjustment.** Bars show fgsea NES for CCD (top panels) and ICD (bottom panels) in each cohort, before (left bars) and after (right bars) adding MuSiC-derived composition covariates to the DE model. The sign and magnitude of NES are preserved after adjustment, supporting composition-robust subtype biology.

We re-fit bulk differential expression with and without composition covariates (centered EPI/IMM/STR) and ran GSEA on the ICD and CCD gene sets using Wald statistics as ranks. Normalized enrichment scores (NES) and adjusted p-values were compared before vs. after adjustment. In both cohorts, the directionality of enrichment persisted after accounting for composition (Fig. 11). This indicates that the subtype-associated pathways are not artifacts of varying tissue mixture but reflect composition-independent transcriptional programs (see Fig. 12).

Together, (i) recovery of subtype modules in the expected organ–compartment contexts at single-cell resolution, (ii) biologically plausible bulk tissue compositions, and (iii) persistence of ICD/CCD pathway enrichment after composition adjustment, provide orthogonal validation that our subtype signals are genuine and not confounded by sampling of different cell mixtures. These analyses support the central claim that ICD is characterized by ileum-biased stromal/immune transcriptional activity, whereas CCD is driven by colon-biased epithelial programs.

## 4 Discussion

Together, the transcriptomic, proteomic, and single-cell analyses converge on a consistent model: ileal Crohn’s disease is characterized by epithelial metabolic stress and impaired barrier energetics, whereas colonic Crohn’s disease is dominated by innate immune activation and cytokine-driven inflammation. These mechanistic differences offer a rationale for why anatomically defined subtypes respond differently to standard biologic therapies and underscore the need for molecularly informed treatment strategies. Although CKB and SPP1 emerged as representative hubs within their respective networks, they should be interpreted not as singular therapeutic targets but as anchors of broader pathway modules. The framework established here includes combining bulk RNA-seq, network analysis, and multi-omic validation. This provides a template for identifying subtype-specific biology, but future studies will be required to evaluate the clinical utility of these molecular signatures.

## 5 Limitations

The conclusions in this study should be interpreted within the context of several methodological boundaries. The analysis relies on cross-sectional bulk RNA-seq cohorts, which provide high statistical power but only capture a single molecular timepoint per patient. The multi-omic validation (proteomic enrichment, single-cell module recovery, and composition-adjusted modeling) support the subtype conclusions to an extent. Longitudinal sampling would determine how these molecular signatures evolve during treatment, remission, or flare. This would strengthen the mechanistic interpretation of subtype-specific pathways but are not necessary for identifying robust differences between ICD and CCD in the present framework.

Bulk RNA-seq inherently blends signals across epithelial, immune, and stromal cell type compartments. While MuSiC-based deconvolution and covariate-adjusted differential expression reduce cell-type composition effects, single-cell validation cannot reconstruct the microenvironment of every sample. Consequently, some pathway differences may reflect both true transcriptional regulation and biologically meaningful shifts in tissue composition.

Protein–protein interaction networks also depend on the completeness of curated interaction databases. Centrality metrics such as degree or betweenness may reflect annotation density rather than absolute biological hierarchy. Nevertheless, the consistency of CKB- and SPP1-anchored modules across transcriptomic, proteomic, and single-cell contexts indicates that these hubs represent reproducible components of subtype biology.

Finally, clinical variables across public datasets are inconsistently reported, limiting how much factors like medication exposure, disease duration, or age could be incorporated into the models. Integrating clinical metadata with multi-omic profiling would improve translational relevance of the present molecular findings.

Overall, these limitations reflect areas for future improvement. The core result is the same: ileal and colonic Crohn’s disease exhibit reproducible, multi-omic, and biologically distinct molecular architectures.

## 6 Conclusions

This study integrates bulk RNA-seq, protein–protein interaction networks, and multi-omic validation to define molecular signatures distinguishing ileal and colonic Crohn’s disease. By mapping DEGs onto BioGRID networks and validating pathway directionality through proteomic and single-cell datasets, we demonstrate that ICD is driven by epithelial metabolic stress while CCD is driven by innate immune activation. These findings expand upon prior transcriptomic work by mapping subtype-specific regulatory hubs and pathways validated across independent omic layers. The resulting subtype signatures provide a framework for further sub classification and nominate CKB- and SPP1-centered modules as testable targets. This multi-omic approach highlights the importance of moving beyond anatomical labels toward molecularly informed therapeutic strategies in Crohn’s disease.

## Declaration of competing interest

The authors declare that they have no known competing financial interests or personal relationships that could have appeared to influence the work reported in this paper.

## Data Availability

Data will be made available on request.

## Appendix A Supplementary data

MMC S1 Survey of prior IBD/Crohn’s subtype studies, their cohorts, methods, and limitations. MMC S1

MMC S2 Identified ICD and CCD DEGs. MMC S2

MMC S3 . MMC S3

MMC S4 . MMC S4

MMC S5 . MMC S5

MMC S6 . MMC S6
